# Possibilities for preventive and personalized therapy of cancer by drugs screening

**DOI:** 10.1186/1878-5085-5-S1-A34

**Published:** 2014-02-11

**Authors:** Igor V Volchek, Tamara V Sologub, Andrei S Petrov

**Affiliations:** 1 DiscoveryMed Ltd, St. Petersburg, Russia; 2 Research Institute of Influenza, St. Petersburg, Russia

## 

The purpose of this study was the search of new biomarkers for preventive and personalized therapy of cancer. Thiol-disulfide system was selected as a target for drug screening as the most important biochemical system that characterizes the state of the antioxidant defense and redox processes. The study involved more than 1,200 patients with chronic hepatitis C (HCV), genital herpes, human papillomavirus infection, endometriosis, non-small cell lung cancer (NSCLC), metastatic kidney and prostate cancer. An original method for screening drug preparations was proposed, where whole blood with anticoagulant (EDTA) was incubated in thermostat (37°C) in the presence of drugs for 1 h, the control samples incubated with saline [1]. Determination of SH-and SS-groups in hemolysate was done by spectrophotometry. In processing the data performance SH-groups and SH/SS ratio in the control and experimental samples were compared. It was found that all medications, including cytostatic, hormonal, antiviral and immune have either a stimulating or a depressing effect on blood thiol-disulfide system, by which one can predict the efficacy and safety of therapy, and to individualize treatment [2-4]. In two controlled studies a 3-fold increase in the incidence of virological response of personalized monotherapy with interferon (IFN) of HCV patients was shown, and the rate of adverse effects was decreased by 6 times compared with standard IFN therapy. Prognostic significance of SH/SS -test for the treatment of HCV patients was 89.8% [3]. Control studies also demonstrated a 2-fold increase in the efficiency of personalized hormonal therapy of endometriosis compared with standard and a 2-fold increase in the frequency of partial remission rate of NSCLC after personalized treatment by cytostatics compared with standard chemotherapy [4]. Thus, the thiol-disulfide system can be regarded as a universal biomarker for preventive and personalized therapy of cancer. The proposed method of drugs screening using SH/SS-test can be used to personalize therapy of viral diseases and cancer by hormonal, antiviral, cytostatic and immune preparations to improve efficiency and overcome resistance to drugs, finding the best drugs, their doses and combinations, reducing the frequency of side effects and complications to prevent cancer, its relapse and progression.
